# Understanding Cognitive Heterogeneity in Children Referred to Mental Health Services: Evidence from a Large Clinical Sample

**DOI:** 10.3390/children13101365

**Published:** 2026-10-08

**Authors:** Selçuk Özkan, Abdullah Ayaz

**Affiliations:** Department of Child and Adolescent Psychiatry, Faculty of Medicine, Kütahya Health Sciences University, Kütahya 43100, Türkiye; abdullah.ayaz@ogr.ksbu.edu.tr

**Keywords:** child and adolescent psychiatry, cognitive assessment, cognitive heterogeneity, neurodevelopment, clustering, clinical sample

## Abstract

**Highlights:**

**What are the main findings?**

- Cognitive variation was dominated by a general factor; a smaller verbal–performance contrast closely tracked the conventional PIQ-VIQ discrepancy.
- After the general cognitive level was removed, full- and equal-covariance mixture models selected one class, and forced cluster solutions showed weak separation.

**What are the implications of the main findings?**

- Within these ten WISC-R subtests, the available evidence favoured graded variation over clearly separated profile-based subtypes.
- Isolated profile differences should be interpreted in relation to the overall cognitive level and the child’s broader developmental and clinical context.

**Abstract:**

**Background**: General ability and verbal–performance organisation are established features of Wechsler batteries, while the diagnostic value of subtest profile types remains contested. We examined whether ten WISC-R subtests in a large clinical referral sample contained reproducible profile-based groups beyond the overall cognitive level. **Methods**: Ten WISC-R subtests from 1261 children in a single-centre child psychiatry archive were analysed using principal components, parallel analysis, an orthogonal bifactor model, Gaussian mixtures with multiple covariance structures, k-means, level-free profile analyses, and 500 subsample stability replications. **Results**: The first component accounted for 50.4% of the variance and correlated with Full-Scale IQ (r = 0.997). The second accounted for 11.0% and closely tracked PIQ-VIQ (r = 0.942); the parallel analysis retained only the first component. A bifactor model showed a strong general factor (omega-hierarchical = 0.814; explained common variance = 0.767). Raw-score k-means produced a reproducible higher-versus-lower level split (mean Full-Scale IQ 101.4 vs. 71.6; silhouette = 0.261), but, after removing the general level, the full- and equal-covariance mixture models selected one class, and forced k-means solutions had silhouettes of 0.08–0.12. **Conclusions**: Within the ten WISC-R variables examined, no clearly separated profile-based subtypes were detected beyond graded differences in the general level and verbal–performance balance. These findings concern the internal structure of one battery in one clinical referral sample and do not establish the absence of clinically meaningful subtypes defined by broader functional measures.

## 1. Introduction

Diagnostic categories are central to communication, treatment planning, and the organisation of services in child and adolescent mental health. However, the difficulties seen in routine practice often extend across diagnostic boundaries. Attention, learning, language, motor, social communication, and broader cognitive difficulties frequently occur together, while children with the same diagnosis may have markedly different strengths and needs. Contemporary transdiagnostic models therefore describe neurodevelopmental characteristics as overlapping dimensions that extend from typical functioning to clinically significant difficulty [1]. Work in learning disorders has reached a similar conclusion: categorical thresholds remain useful, but much of the variation underlying children’s difficulties may be continuous [2].

This distinction matters in everyday clinical assessment. If cognitive variation is organised into reproducible subtypes, recognising these patterns could help clinicians to anticipate needs and select assessments or interventions. If the apparent groups mainly reflect divisions along a continuous range of general cognitive functioning, assigning subtype labels may add little and may obscure individual differences. In a heterogeneous group of 530 children struggling at school, Astle and colleagues identified four data-driven cognitive profiles, but profile membership was not predicted by the diagnosis or referral reason [3]. A subsequent transdiagnostic study of 1529 children and adolescents similarly found limited correspondence between cognitive clusters and traditional diagnostic status [4]. These findings support the examination of cognitive performance across the clinical population, rather than beginning with preselected diagnostic groups.

These questions have a substantial history in Wechsler interpretation. The dominant general factor and the verbal–performance organisation of the WISC-R were documented in early factor-analytic work [5]. Normative core profile studies later showed that empirically derived profile types were strongly influenced by the overall level [6], while reviews and clinical studies found limited reliability and diagnostic utility for subtest scatter and shape-based interpretation [7,8]. This literature makes the relevant question narrower: not whether Wechsler subtests share a general factor, but whether a clinical referral sample contains reproducible profile shape groups after the general level is removed.

Cognitive tests collected during routine child psychiatry care provide an opportunity to study this question in real-world settings. Individual subtests sample different task demands, but their interpretation must remain anchored in the child’s overall level of performance, developmental history, referral context, and daily functioning. Cognitive ability is meaningfully associated with school achievement [9], yet a cognitive score or an uneven profile is not diagnostically specific. Associations between cognitive functioning and emotional symptoms may also be small and dependent on the measure and informant [10,11]. Clinical archives also require explicit quality checks because data recorded for care may be incomplete, inconsistent, or selective when repurposed for research [12].

The present study therefore re-examined an established psychometric question in a large Turkish child psychiatry referral archive. Rather than treating a dominant general factor or verbal–performance contrast as novel, we asked whether complementary modern methods would identify reproducible profile shape groups beyond the overall level. The study extends the established literature by combining a large clinical referral sample from an underrepresented setting with explicit comparisons of covariance structures, level-free profiles, stability, and common-factor models. Candidate groups were considered potentially meaningful only when supported jointly by fit, separation, stability, non-trivial size, and interpretable shape differences [13,14].

## 2. Materials and Methods

### 2.1. Study Design and Setting

This single-centre, retrospective, cross-sectional study used cognitive assessments administered during routine care in a university-affiliated child and adolescent psychiatry clinic. Assessments were requested by treating clinicians according to individual clinical need; referral indications and pathways were not recorded in a sufficiently standardised form for analysis. Diagnostic labels in routine records were also incomplete and heterogeneous, so disorder-specific proportions and diagnostic validation analyses were not included. The study is reported in accordance with the STROBE and RECORD recommendations [15,16]. The unit of analysis was the child rather than test administration. For children with more than one assessment, the first eligible assessment, corresponding to the youngest eligible age, was used. Direct identifiers were removed, and each child was represented by a study identifier.

### 2.2. Cohort Construction and Data Quality

The archive contained 2049 test records from 1900 unique children. Eligibility was restricted to children aged 6–16 years. Verbal IQ, Performance IQ, and Full-Scale IQ were required to be available and greater than zero. All ten subtest scaled scores also had to be present and fall within the interpretable range of 1–19.

During the initial preparation, zero values had been treated as falling within an allowable 0–19 range. Before analysis, we examined the distribution of these values and found that zeros were concentrated particularly in the Similarities subtest and occurred in patterns consistent with missing or unadministered subtests. Zero values were therefore treated as missing or invalid rather than as observed performance. Following these quality checks, 1261 children were included in the main cognitive analysis.

### 2.3. Cognitive Measures

The analysis included five verbal subtests (Information, Similarities, Arithmetic, Comprehension, and Digit Span) and five performance subtests (Picture Completion, Picture Arrangement, Block Design, Object Assembly, and Coding) from the Wechsler Intelligence Scale for Children—Revised (WISC-R). Because scaled scores are age-standardised, they were not adjusted again for age. Subtest scores were standardised within the study sample before multivariable analysis. Composite IQ scores were not used to form dimensions or groups; they were retained only to support the clinical interpretation of the resulting patterns.

### 2.4. Analysis of Cognitive Structure

We first used principal component analysis (PCA) on sample-standardised subtest scores. We report eigenvalues, explained variance, eigenvector coefficients, and conventional component loadings. Horn parallel analysis used 1000 independently permuted datasets and the 95th percentile of their eigenvalues. The second component was compared directly with PIQ-VIQ. We additionally fitted orthogonal one-factor and bifactor common-factor models to the subtest correlation matrix. The bifactor model included a general factor loading on all subtests and verbal and performance group factors loading on their respective five subtests. Model fit was summarised using the CFI, TLI, RMSEA, and SRMR; omega-hierarchical and explained common variance quantified general-factor dominance [17,18].

We used Gaussian mixture models and k-means to examine candidate groups. Mixture models with one to ten classes were fitted using full, equal (tied), diagonal, and spherical covariance structures; the BIC, AIC, entropy, silhouette, and smallest class size were reported [19]. K-means solutions containing two to six clusters were evaluated using the silhouette coefficient [20]. To test profile shape directly, clustering was repeated after (i) removing the first principal component and restandardising the residuals and (ii) ipsatising each child’s profile by subtracting the within-person subtest mean and restandardising the columns. Stability was estimated in 500 random 80% subsamples. For each subsample, the adjusted Rand index compared the subsample solution with the full-sample assignments for the same children. Pairwise correlations among mean raw subtest profiles were reported for k = 2–6. All analyses used multiple initialisations and fixed random seeds. These expanded analyses were conducted in response to peer review; no single statistic was treated as proof of a categorical structure.

### 2.5. Software

Analyses were conducted in Python 3.12.13 using NumPy 2.3.5, pandas 2.2.3, SciPy 1.17.0, and scikit-learn 1.8.0. The cognitive analyses were descriptive and unsupervised; inferential *p* values were not used to select component or cluster solutions. The reproducible analysis code is provided as Supplementary File S2.

## 3. Results

### 3.1. Cohort Characteristics and Data Quality

**Figure 1:**
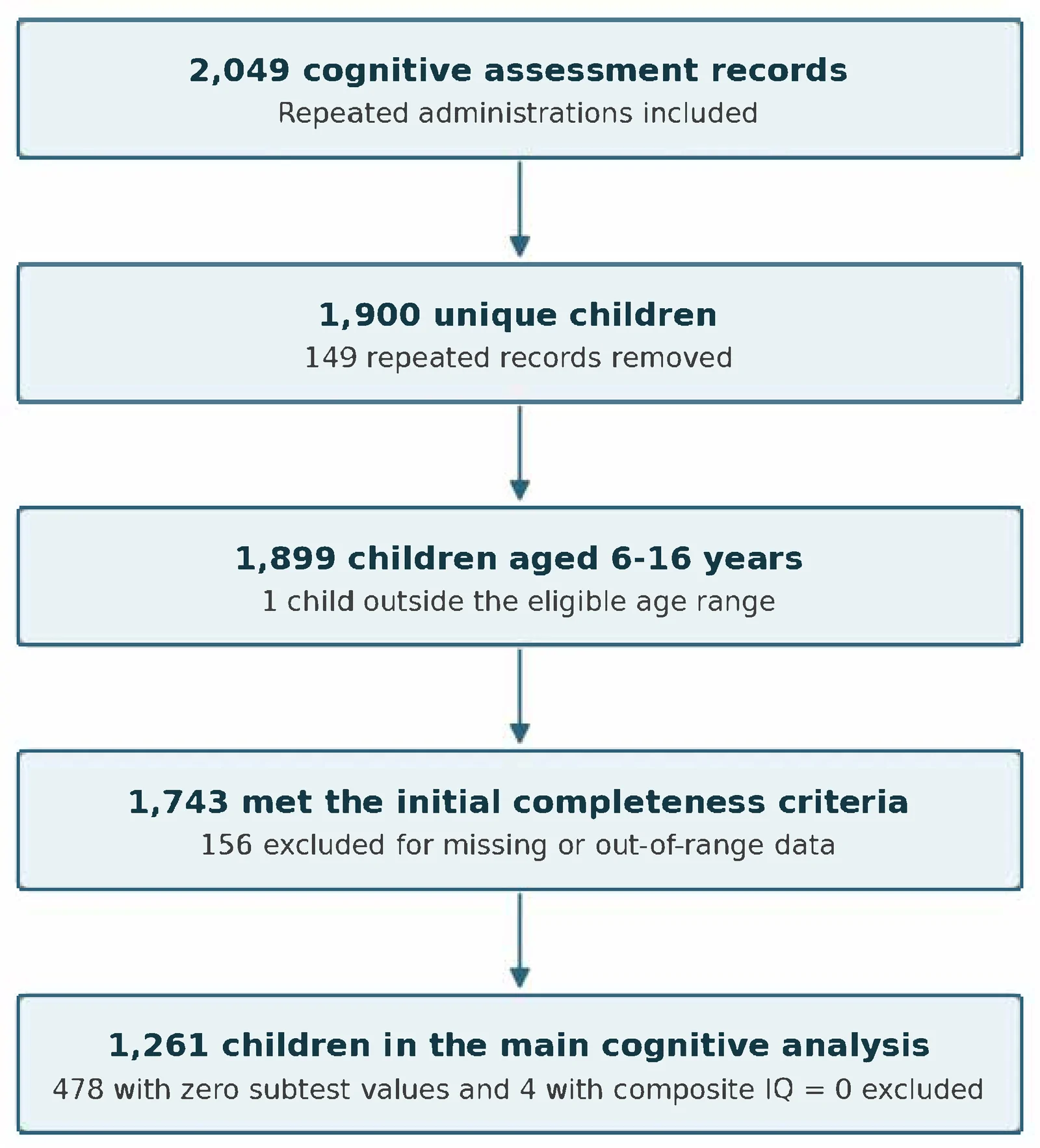
Flow of records and children through cohort selection. Note: Zero subtest scores were treated as invalid because the data quality review showed a pattern consistent with missing or unadministered subtests.

The main analysis included 1261 children. The mean age was 9.65 ± 2.25 years (range, 6.00–15.51), and 859 children (68.1%) were male. Of the 1743 children who met the initial completeness criteria, 478 were excluded after zero subtest scores were reclassified as missing or invalid; four additional children with a composite IQ value of zero were excluded. The cohort selection process is presented in Figure 1, and the characteristics of the final sample are summarised in Table 1.

### 3.2. Main Dimensions of Cognitive Performance

The first component accounted for 50.4% of the subtest variance (eigenvalue = 5.04). Its eigenvector coefficients ranged from 0.287 to 0.360 and its component loadings from 0.645 to 0.809 (Table 2). It correlated with Full-Scale IQ (r = 0.997), Verbal IQ (r = 0.915), and Performance IQ (r = 0.903). These associations were expected because all quantities were derived from the same battery; they describe how the multivariable solution relates to conventional composites, rather than providing independent validation.

The second component accounted for 11.0% of the variance (eigenvalue = 1.10). It correlated negatively with Verbal IQ (r = −0.380), positively with Performance IQ (r = 0.402), and very strongly with PIQ-VIQ (r = 0.942). Its near-zero correlation with Full-Scale IQ (r = 0.018) was therefore interpreted cautiously: PCA components are orthogonal by construction, and the first component was almost identical to Full-Scale IQ. The parallel analysis retained only the first component because the second observed eigenvalue (1.101) was below the 95th-percentile random eigenvalue (1.128).

The one-factor common-factor model showed an inadequate fit (CFI = 0.881, TLI = 0.847, RMSEA = 0.122, SRMR = 0.068). The orthogonal bifactor model fitted substantially better (CFI = 0.988, TLI = 0.978, RMSEA = 0.046, SRMR = 0.021). Omega-hierarchical was 0.814, omega-total was 0.910, and the explained common variance for the general factor was 0.767. Group factor loadings were smaller and less consistent for verbal subtests than performance subtests (Tables S6 and S7).

### 3.3. Evaluation of Candidate Cognitive Subtypes

The mixture model results depended strongly on the covariance specification. In the raw standardised scores, the BIC selected four classes with full covariance and three with equal covariance, whereas the diagonal and spherical models selected eight classes. Separation remained weak: the silhouettes for the selected raw-score solutions ranged from 0.049 to 0.149. This covariance sensitivity supports the concern that diagonal models can create ordered classes to reproduce within-class correlations. Full results for k = 1–10, including the entropy and smallest class size, are provided in Table S3.

Raw-score k-means again favoured a two-cluster solution, but its silhouette was low (0.261). Across 500 80% subsamples, the median adjusted Rand index was 0.984 (95% empirical interval, 0.884–1.000). The groups mainly differed in general level: the mean Full-Scale IQ was 101.37 versus 71.59, and their raw mean subtest profiles correlated at r = 0.929. For k = 3–6, the mean pairwise profile correlations declined from 0.857 to 0.488, but separation and stability also decreased (Tables S4 and S5). After PC1 residualisation, the full- and equal-covariance mixture models selected one class across the k = 1–10 search. The same occurred after ipsatisation. Forced two-cluster k-means solutions were reproducible (median ARI = 0.949 and 0.930, respectively) but poorly separated (silhouette = 0.125 and 0.123), consistent with a stable algorithmic split of a weak continuous contrast, rather than evidence of a natural boundary (Table 3).

## 4. Discussion

### 4.1. Principal Findings

In this large Turkish child psychiatry referral archive, the dominant general factor and verbal–performance organisation were consistent with long-established features of Wechsler batteries. The study’s more specific contribution was to test profile shape groups directly after accounting for the general level and to examine their robustness across complementary models. These analyses did not identify a clearly separated solution: appropriate-covariance mixture models selected one class after level removal, and forced shape clusters showed weak separation. Thus, no clearly separated profile-based subtypes were detected within these ten subtests and this referral sample.

### 4.2. Continuous Cognitive Variation in Clinical Care

The dominant general factor was expected from the positive manifold of Wechsler subtests and Kaufman’s early WISC-R factor analyses [5]. Within the present study, PCA quantified the dominance of this dimension, characterised the secondary verbal–performance contrast, and provided an explicit transformation for testing profile shape after the removal of the general level. It was not interpreted as a new discovery or external validation of g. The parallel analysis retained one component, and the bifactor model provided convergent common-factor evidence: the fit was good (CFI = 0.988, TLI = 0.978, RMSEA = 0.046, SRMR = 0.021), omega-hierarchical was 0.814, and the general factor explained 76.7% of the common variance. The second principal component closely tracked PIQ-VIQ, and its near-zero association with Full-Scale IQ was largely a consequence of orthogonality rather than independent validation.

This interpretation is consistent with the historical WISC profile literature. McDermott and colleagues’ normative core-profile typologies were strongly influenced by overall elevation, and subsequent work questioned the reliability, diagnostic accuracy, and incremental utility of subtest scatter and profile shape [6,7,8]. The present study does not claim the first demonstration of general-level dominance; its contribution lies in evaluating this question in a large clinical referral sample with complementary covariance, stability, common-factor, and level-free analyses. Modern transdiagnostic studies using broader batteries can still identify useful person-centred patterns [3,4,21], but those patterns address a richer construct space than the ten subtests studied here.

Other person-centred studies have identified several cognitive profiles among children with neurodevelopmental difficulties [15]. Such findings do not necessarily conflict with the present results. This archive contained ten subtests from one test family, all of which share variance related to general cognitive ability. Studies that combine direct measures of language, learning, executive functioning, social cognition, adaptive functioning, and motor skills may identify clinically useful patterns that are not visible in a conventional intelligence test profile. The current conclusion is therefore deliberately limited: these ten subtests did not independently support sharply separated cognitive subtypes in this clinical sample.

### 4.3. Clinical Interpretation

The dataset did not contain standardised diagnoses, symptoms, achievement, adaptive functioning, or outcomes and therefore could not test disorder-specific or predictive validity. Nevertheless, the large clinical referral sample supports a practical interpretive implication: isolated subtest discrepancies and profile labels should be considered in relation to the child’s overall cognitive level rather than treated as self-sufficient clinical entities. Individual strengths and weaknesses may remain clinically informative, but diagnostic and support decisions also require the direct assessment of developmental history, academic skills, adaptive functioning, symptoms, and everyday needs.

Cognitive and emotional processes may be related in children with neurodevelopmental difficulties, but these associations are often modest and sensitive to how both domains are measured [22]. The absence of diagnosis-specific or symptom-specific analyses in the present study should therefore not be taken to mean that clinical presentation is irrelevant. Rather, the results define the broad cognitive structure that should be considered before more specific clinical associations are examined with standardised measures.

### 4.4. Strengths and Limitations

The study has several strengths. It examined a large sample drawn from routine child psychiatry care, used unique children rather than repeated test administrations as the unit of analysis, and evaluated cognitive structure without imposing diagnostic groups in advance. Candidate clusters were assessed using complementary indicators rather than a single model statistic, and their profile shapes were considered alongside statistical fit and stability.

Several limitations require emphasis. The study was retrospective and conducted at one centre among clinically referred children; the findings should not be generalised to the general population. The Turkish WISC-R norms were established in an earlier period, which primarily limits the interpretation of absolute IQ values and the reported cluster means as current population estimates [23]. The present analyses, however, focused chiefly on the relative covariance and profile structure within the same clinical sample, which are less directly affected by the norm age than absolute score levels. Contemporary Wechsler scales distinguish working memory and processing speed more explicitly, and broader neuropsychological batteries sample additional domains; these instruments may therefore refine the group factor patterns without rendering the present within-sample structural findings uninformative. Replication with contemporary measures remains valuable. The first component and Full-Scale IQ also share the same subtest information, and their correlation is not external validation.

The reduction from 1743 initially eligible records to 1261 complete analytical records is a potentially important source of selection bias. The excluded group was defined by at least one zero subtest score, which the archive could not distinguish reliably among non-administration, incomplete testing, data entry convention, or extremely limited performance. These exclusions were therefore not assumed to be random. Missing or unadministered subtests may be more common among children with greater developmental, behavioural, communication, or attentional difficulties; limited test engagement; fatigue; or assessments adapted to clinical circumstances. Complete-case analysis may under-represent children for whom standard administration was most difficult and could make the observed structure appear more orderly. The archive lacked standardised variables needed to determine the direction or magnitude of this bias.

The component and cluster solutions depend on the available variables and analytical choices, and other test batteries may reveal different patterns. Diagnostic status, academic achievement, adaptive functioning, treatment exposure, and longitudinal outcomes were not available in a sufficiently standardised form for inclusion. The clinical meaning and predictive value of the observed dimensions therefore remain to be established. Finally, the cognitive structure was not evaluated in an independent sample.

## 5. Conclusions

Among children referred to one child psychiatry clinic, a dominant general factor accounted for most common variance across ten WISC-R subtests. The secondary principal component largely reproduced the conventional PIQ-VIQ discrepancy. After the general level was removed, appropriate-covariance mixture models selected one class, and forced profile clusters were weakly separated. Thus, no clearly separated profile-based subtypes were detected using the variables and methods available. Clinically, individual profile differences may still inform assessment, but they should be interpreted in relation to the overall cognitive level and the child’s broader developmental, academic, adaptive, and psychiatric context. The study does not show that clinically meaningful subtypes defined by broader measures are absent; replication with contemporary batteries and external clinical criteria remains warranted.

## Figures and Tables

**Table 1:** Characteristics of the main analytical sample.

Characteristic	Value
Children included, *n*	1261
Age, mean ± SD, years	9.65 ± 2.25
Age range, years	6.00–15.51
Male, *n* (%)	859 (68.1%)

SD, standard deviation.

**Table 2:** Subtest contributions to the first two cognitive components.

Subtest	PC1 Eigenvector Coefficient	PC2 Eigenvector Coefficient	Component 2 Direction
Information	0.360	−0.235	Verbal
Similarities	0.299	−0.235	Verbal
Arithmetic	0.336	−0.319	Verbal
Comprehension	0.310	−0.258	Verbal
Digit Span	0.304	−0.399	Verbal
Picture Completion	0.308	0.355	Performance
Picture Arrangement	0.327	0.185	Performance
Block Design	0.331	0.339	Performance
Object Assembly	0.295	0.528	Performance
Coding	0.287	0.089	Performance

Note: Values are eigenvector coefficients, not subtest component correlations. Conventional component loadings, all eigenvalues, the parallel analysis thresholds, and the full subtest correlation matrix are provided in Tables S1 and S2. Component signs may be reversed without changing the solution.

**Table 3:** Summary of clustering and level-free sensitivity analyses.

Analysis	Selected/Forced K	Criterion	Separation	Stability or Class Size
Raw k-means	2	Largest silhouette	0.261	Median ARI 0.984
PC1-residual k-means	2 (forced)	Shape sensitivity	0.125	Median ARI 0.949
Ipsatised k-means	2 (forced)	Shape sensitivity	0.123	Median ARI 0.930
Raw GMM, full covariance	4	Minimum BIC	Silhouette 0.061	Smallest n = 156
Raw GMM, equal covariance	3	Minimum BIC	Silhouette 0.149	Smallest n = 245
PC1-residual GMM, full/equal	1	Minimum BIC	Not applicable	n = 1261
Ipsatised GMM, full/equal	1	Minimum BIC	Not applicable	n = 1261

Note: GMM, Gaussian mixture model; ARI, adjusted Rand index. Stability estimates used 500 random 80% subsamples. Complete k = 1–10 results, entropy, smallest class size, and k = 2–6 profile correlations are reported in Tables S3–S5.

## Data Availability

Participant-level data are not publicly available because they contain sensitive clinical information and are subject to privacy protections. De-identified data may be made available by the corresponding author upon reasonable scientific request and required ethical and institutional approvals. A reproducible analysis code is provided as Supplementary File S2.

## Supplementary Materials

The following supporting information can be downloaded at https://www.mdpi.com/article/10.3390/children13101365/s1: Table S1, Pearson correlations among the ten WISC-R subtests; Table S2, PCA eigenvalues and Horn parallel analysis; Table S3, Gaussian mixture model comparisons across data representations, covariance structures, and k = 1–10; Table S4, K-means separation and stability across 500 random 80% subsamples; Table S5, Pairwise correlations among raw mean subtest profiles for k = 2–6; Table S6, Standardised loadings from the orthogonal bifactor model; Table S7, Common-factor model fit and general-factor indices; Supplementary File S2, Reproducible Python analysis code.
