# Supplementary material for: Understanding Cognitive Heterogeneity in Children Referred to Mental Health Services: Evidence from a Large Clinical Sample
Source: Children (Basel). 2026 Oct 8;13(10):1365. doi: 10.3390/children13101365 (PMC13703743; doi:10.3390/children13101365)
Supplement: Supplementary file 1 [file children-13-01365-s001.zip › children-4580921-Supplementary-S1.pdf]

Supplementary File S1

Understanding Cognitive Heterogeneity in Children Referred to Mental Health Services: Evidence from a Large Clinical Sample

Selçuk Özkan and Abdullah Ayaz

Table S1. Pearson correlations among the ten WISC-R subtests

Subtest	Information	Similarities	Arithmetic	Comprehension	Digit Span	Picture Completion	Picture Arrangement	Block Design	Object Assembly	Coding
Information	1.000	0.543	0.640	0.571	0.574	0.473	0.517	0.498	0.400	0.452
Similarities	0.543	1.000	0.468	0.493	0.419	0.371	0.415	0.410	0.323	0.325
Arithmetic	0.640	0.468	1.000	0.492	0.611	0.378	0.450	0.476	0.343	0.434
Comprehension	0.571	0.493	0.492	1.000	0.456	0.416	0.410	0.375	0.336	0.383
Digit Span	0.574	0.419	0.611	0.456	1.000	0.325	0.420	0.386	0.284	0.355
Picture Completion	0.473	0.371	0.378	0.416	0.325	1.000	0.512	0.511	0.548	0.379
Picture Arrangement	0.517	0.415	0.450	0.410	0.420	0.512	1.000	0.508	0.490	0.462
Block Design	0.498	0.410	0.476	0.375	0.386	0.511	0.508	1.000	0.631	0.457
Object Assembly	0.400	0.323	0.343	0.336	0.284	0.548	0.490	0.631	1.000	0.369
Coding	0.452	0.325	0.434	0.383	0.355	0.379	0.462	0.457	0.369	1.000

Table S2. PCA eigenvalues and Horn parallel analysis

Component	Eigenvalue	Variance %	Parallel mean	Parallel 95th	Retained
1	5.042	50.383	1.143	1.182	True
2	1.101	11.005	1.100	1.128	False
3	0.691	6.904	1.067	1.091	False
4	0.619	6.190	1.038	1.059	False
5	0.544	5.435	1.011	1.032	False
6	0.515	5.148	0.986	1.004	False
7	0.437	4.371	0.960	0.979	False
8	0.390	3.898	0.933	0.954	False
9	0.335	3.345	0.903	0.927	False
10	0.333	3.323	0.866	0.895	False

Table S3. Gaussian mixture model comparisons across data representations, covariance structures, and k = 1-10

Dataset	Covariance	K	BIC	AIC	Entropy	Silhouette	Smallest class n	Smallest class %
Raw	full	1	30729.079	30395.001	-	-	1261	100.000
Raw	full	2	30833.397	30160.102	0.601	0.219	594	47.105
Raw	full	3	31042.461	30029.948	0.689	0.109	353	27.994
Raw	full	4	29780.172	28428.441	0.750	0.061	156	12.371
Raw	full	5	30141.826	28450.878	0.742	0.057	156	12.371
Raw	full	6	30460.387	28430.221	0.792	0.047	152	12.054
Raw	full	7	30786.798	28417.415	0.797	0.030	86	6.820
Raw	full	8	31071.021	28362.420	0.832	0.019	66	5.234
Raw	full	9	31405.954	28358.136	0.828	0.020	45	3.569
Raw	full	10	31729.397	28342.361	0.845	0.008	48	3.807
Raw	tied	1	30729.079	30395.001	-	-	1261	100.000
Raw	tied	2	30784.712	30394.097	0.472	0.241	569	45.123
Raw	tied	3	30711.912	30264.761	0.659	0.149	245	19.429
Raw	tied	4	30729.512	30225.825	0.611	0.108	183	14.512
Raw	tied	5	30749.566	30189.343	0.629	0.100	82	6.503
Raw	tied	6	30758.801	30142.042	0.690	0.087	105	8.327
Raw	tied	7	30783.564	30110.268	0.691	0.082	64	5.075
Raw	tied	8	30854.159	30124.327	0.672	0.064	76	6.027
Raw	tied	9	30880.699	30094.331	0.699	0.064	48	3.807
Raw	tied	10	30938.488	30095.584	0.704	0.054	31	2.458
Raw	diag	1	35928.423	35825.630	-	-	1261	100.000
Raw	diag	2	32484.399	32273.673	0.855	0.259	625	49.564
Raw	diag	3	31469.713	31151.054	0.862	0.178	240	19.033
Raw	diag	4	31270.703	30844.111	0.805	0.131	194	15.385
Raw	diag	5	31062.963	30528.439	0.799	0.123	151	11.975
Raw	diag	6	31034.698	30392.241	0.776	0.103	154	12.213
Raw	diag	7	31026.255	30275.864	0.775	0.095	140	11.102
Raw	diag	8	29778.236	28919.913	0.798	0.049	107	8.485
Raw	diag	9	31135.631	30169.375	0.790	0.086	51	4.044
Raw	diag	10	29900.386	28826.197	0.799	0.049	76	6.027
Raw	spherical	1	35864.166	35807.630	-	-	1261	100.000
Raw	spherical	2	32509.946	32391.733	0.846	0.260	621	49.247

Dataset	Covariance	K	BIC	AIC	Entropy	Silhouette	Smallest class n	Smallest class %
Raw	spherical	3	31510.061	31330.173	0.846	0.180	261	20.698
Raw	spherical	4	31301.959	31060.395	0.771	0.134	215	17.050
Raw	spherical	5	31133.459	30830.219	0.767	0.129	152	12.054
Raw	spherical	6	31072.316	30707.400	0.748	0.121	149	11.816
Raw	spherical	7	31007.203	30580.611	0.743	0.106	134	10.626
Raw	spherical	8	30997.991	30509.723	0.753	0.098	72	5.710
Raw	spherical	9	31003.765	30453.821	0.741	0.091	75	5.948
Raw	spherical	10	31008.791	30397.171	0.741	0.082	57	4.520
PC1 residual	full	1	18072.192	17738.114	-	-	1261	100.000
PC1 residual	full	2	18391.361	17718.066	0.365	0.092	602	47.740
PC1 residual	full	3	18700.011	17687.498	0.454	0.052	328	26.011
PC1 residual	full	4	19071.556	17719.826	0.524	0.045	226	17.922
PC1 residual	full	5	19392.672	17701.723	0.564	0.032	208	16.495
PC1 residual	full	6	19733.271	17703.105	0.601	0.026	153	12.133
PC1 residual	full	7	20060.647	17691.264	0.672	0.030	112	8.882
PC1 residual	full	8	20424.218	17715.617	0.693	0.013	73	5.789
PC1 residual	full	9	20750.391	17702.573	0.716	0.004	87	6.899
PC1 residual	full	10	21086.599	17699.563	0.739	0.001	66	5.234
PC1 residual	tied	1	18072.192	17738.114	-	-	1261	100.000
PC1 residual	tied	2	18151.148	17760.533	0.306	0.119	519	41.158
PC1 residual	tied	3	18168.383	17721.232	0.400	0.073	358	28.390
PC1 residual	tied	4	18208.773	17705.086	0.438	0.075	153	12.133
PC1 residual	tied	5	18235.569	17675.346	0.485	0.072	87	6.899
PC1 residual	tied	6	18292.350	17675.591	0.473	0.065	79	6.265
PC1 residual	tied	7	18327.703	17654.408	0.544	0.058	72	5.710
PC1 residual	tied	8	18407.431	17677.599	0.487	0.073	84	6.661
PC1 residual	tied	9	18415.349	17628.981	0.560	0.066	53	4.203
PC1 residual	tied	10	18468.622	17625.717	0.571	0.065	42	3.331
PC1 residual	diag	1	35928.423	35825.630	-	-	1261	100.000
PC1 residual	diag	2	35434.552	35223.826	0.575	0.124	552	43.775
PC1 residual	diag	3	35361.241	35042.582	0.566	0.078	333	26.408
PC1 residual	diag	4	35357.012	34930.420	0.576	0.072	245	19.429
PC1 residual	diag	5	35414.084	34879.559	0.579	0.058	214	16.971

Dataset	Covariance	K	BIC	AIC	Entropy	Silhouette	Smallest class n	Smallest class %
PC1 residual	diag	6	35466.497	34824.039	0.637	0.066	88	6.979
PC1 residual	diag	7	35501.539	34751.148	0.647	0.056	133	10.547
PC1 residual	diag	8	35543.054	34684.731	0.664	0.053	100	7.930
PC1 residual	diag	9	35633.317	34667.061	0.677	0.047	70	5.551
PC1 residual	diag	10	35707.025	34632.836	0.698	0.056	44	3.489
PC1 residual	spherical	1	35864.166	35807.630	-	-	1261	100.000
PC1 residual	spherical	2	35402.356	35284.144	0.531	0.125	558	44.251
PC1 residual	spherical	3	35290.958	35111.070	0.513	0.075	344	27.280
PC1 residual	spherical	4	35284.122	35042.558	0.521	0.053	253	20.063
PC1 residual	spherical	5	35282.073	34978.833	0.570	0.034	95	7.534
PC1 residual	spherical	6	35285.177	34920.261	0.573	0.058	96	7.613
PC1 residual	spherical	7	35285.726	34859.134	0.580	0.052	76	6.027
PC1 residual	spherical	8	35280.695	34792.427	0.600	0.050	85	6.741
PC1 residual	spherical	9	35301.064	34751.120	0.625	0.049	64	5.075
PC1 residual	spherical	10	35309.786	34698.166	0.632	0.047	66	5.234
Ipsatised	full	1	18079.840	17745.762	-	-	1261	100.000
Ipsatised	full	2	18395.565	17722.269	0.357	0.090	630	49.960
Ipsatised	full	3	18688.709	17676.196	0.511	0.042	256	20.301
Ipsatised	full	4	19066.573	17714.843	0.564	0.047	250	19.826
Ipsatised	full	5	19411.040	17720.092	0.550	0.027	167	13.243
Ipsatised	full	6	19754.762	17724.596	0.640	0.037	149	11.816
Ipsatised	full	7	20062.561	17693.178	0.621	0.010	131	10.389
Ipsatised	full	8	20399.702	17691.101	0.712	0.026	72	5.710
Ipsatised	full	9	20764.471	17716.652	0.689	-0.002	98	7.772
Ipsatised	full	10	21100.633	17713.597	0.722	0.003	60	4.758
Ipsatised	tied	1	18079.840	17745.762	-	-	1261	100.000
Ipsatised	tied	2	18160.845	17770.231	0.296	0.119	525	41.634
Ipsatised	tied	3	18184.282	17737.132	0.434	0.086	315	24.980
Ipsatised	tied	4	18235.920	17732.233	0.433	0.077	182	14.433
Ipsatised	tied	5	18267.652	17707.429	0.502	0.072	56	4.441
Ipsatised	tied	6	18306.763	17690.004	0.539	0.063	60	4.758
Ipsatised	tied	7	18361.181	17687.886	0.482	0.071	100	7.930
Ipsatised	tied	8	18403.036	17673.204	0.523	0.067	77	6.106

Dataset	Covariance	K	BIC	AIC	Entropy	Silhouette	Smallest class n	Smallest class %
Ipsatised	tied	9	18457.248	17670.880	0.560	0.075	36	2.855
Ipsatised	tied	10	18508.071	17665.167	0.550	0.073	41	3.251
Ipsatised	diag	1	35928.423	35825.630	-	-	1261	100.000
Ipsatised	diag	2	35446.011	35235.285	0.568	0.122	556	44.092
Ipsatised	diag	3	35367.907	35049.248	0.569	0.071	376	29.818
Ipsatised	diag	4	35361.488	34934.896	0.580	0.074	224	17.764
Ipsatised	diag	5	35418.375	34883.850	0.578	0.066	209	16.574
Ipsatised	diag	6	35464.636	34822.178	0.628	0.061	163	12.926
Ipsatised	diag	7	35513.683	34763.292	0.640	0.065	102	8.089
Ipsatised	diag	8	35541.321	34682.998	0.672	0.051	101	8.010
Ipsatised	diag	9	35629.003	34662.747	0.681	0.051	86	6.820
Ipsatised	diag	10	35714.265	34640.076	0.678	0.053	59	4.679
Ipsatised	spherical	1	35864.166	35807.630	-	-	1261	100.000
Ipsatised	spherical	2	35412.589	35294.377	0.526	0.124	555	44.013
Ipsatised	spherical	3	35296.579	35116.691	0.508	0.067	342	27.121
Ipsatised	spherical	4	35293.327	35051.763	0.507	0.053	265	21.015
Ipsatised	spherical	5	35282.160	34978.920	0.562	0.047	86	6.820
Ipsatised	spherical	6	35279.017	34914.101	0.568	0.052	86	6.820
Ipsatised	spherical	7	35291.841	34865.250	0.569	0.063	107	8.485
Ipsatised	spherical	8	35294.564	34806.296	0.589	0.066	89	7.058
Ipsatised	spherical	9	35301.968	34752.025	0.602	0.052	69	5.472
Ipsatised	spherical	10	35329.130	34717.510	0.617	0.054	80	6.344

Table S4. K-means separation and stability across 500 random 80% subsamples

Dataset	K	Silhouette	ARI_mean	ARI_median	ARI_2.5th	ARI_97.5th	Smallest cluster n	Smallest cluster %
Raw	2	0.261	0.969	0.984	0.884	1.000	614	48.692
Raw	3	0.178	0.933	0.968	0.741	0.997	316	25.059
Raw	4	0.138	0.815	0.847	0.609	0.958	258	20.460
Raw	5	0.129	0.796	0.819	0.529	0.961	188	14.909
Raw	6	0.124	0.747	0.757	0.450	0.945	146	11.578
PC1 residual	2	0.125	0.944	0.949	0.886	0.992	591	46.868
PC1 residual	3	0.090	0.729	0.799	0.223	0.962	409	32.435

Dataset	K	Silhouette	ARI_mean	ARI_median	ARI_2.5th	ARI_97.5th	Smallest cluster n	Smallest cluster %
PC1 residual	4	0.085	0.634	0.664	0.235	0.917	285	22.601
PC1 residual	5	0.083	0.437	0.425	0.253	0.761	182	14.433
PC1 residual	6	0.085	0.444	0.410	0.259	0.778	169	13.402
Ipsatised	2	0.123	0.932	0.930	0.868	0.992	603	47.819
Ipsatised	3	0.090	0.718	0.797	0.209	0.955	400	31.721
Ipsatised	4	0.086	0.648	0.677	0.249	0.906	284	22.522
Ipsatised	5	0.084	0.456	0.438	0.218	0.801	223	17.684
Ipsatised	6	0.085	0.471	0.436	0.260	0.782	175	13.878

Table S5. Pairwise correlations among raw mean subtest profiles for k = 2-6

K	Pairwise_profile_r_mean	Pairwise_profile_r_median	Pairwise_profile_r_min	Pairwise_profile_r_max
2.000	0.929	0.929	0.929	0.929
3.000	0.857	0.839	0.819	0.913
4.000	0.629	0.706	0.314	0.858
5.000	0.589	0.773	-0.011	0.927
6.000	0.488	0.596	-0.305	0.942

Table S6. Standardised loadings from the orthogonal bifactor model

Subtest	General	Verbal	Performance	Uniqueness
Information	0.826	0.071	0.000	0.312
Similarities	0.670	-0.087	0.000	0.544
Arithmetic	0.744	0.374	0.000	0.306
Comprehension	0.698	-0.064	0.000	0.509
Digit Span	0.665	0.309	0.000	0.462
Picture Completion	0.554	0.000	0.413	0.522
Picture Arrangement	0.622	0.000	0.308	0.518
Block Design	0.596	0.000	0.499	0.396
Object Assembly	0.470	0.000	0.684	0.312
Coding	0.553	0.000	0.200	0.654

Table S7. Common-factor model fit and general-factor indices

Model	Chi_square	df	CFI	TLI	RMSEA	SRMR	Omega_hierarchical	Omega_total	ECV
one-factor	688.009	35	0.881	0.847	0.122	0.068	-	-	-
bifactor	90.700	25	0.988	0.978	0.046	0.021	0.814	0.910	0.767

Analytical notes

Parallel analysis used 1,000 independently permuted datasets and retained an observed component only when its eigenvalue exceeded the 95th percentile of the corresponding random eigenvalue. Stability analyses used 500 random subsamples containing 80% of children without replacement. Adjusted Rand indices compared each subsample solution with full-sample assignments for the same children. Residual profiles were created by removing PC1 from standardised subtest scores; ipsatised profiles were created by subtracting each child's within-profile mean before restandardising columns.
