# Supplementary material for: Understanding Cognitive Heterogeneity in Children Referred to Mental Health Services: Evidence from a Large Clinical Sample
Source: Children (Basel). 2026 Oct 8;13(10):1365. doi: 10.3390/children13101365 (PMC13703743; doi:10.3390/children13101365)
Supplement: Supplementary file 1 [file children-13-01365-s001.zip › children-4580921-Supplementary-S2.pdf]

Supplementary File S2

Reproducible Python Analysis Code

Understanding Cognitive Heterogeneity in Children Referred to Mental Health Services: Evidence from a Large Clinical Sample

This file contains the reproducible Python analysis code accompanying the article. Participant-level clinical data are not included.

```
import argparse
from pathlib import Path

import numpy as np
import pandas as pd
from scipy.optimize import minimize
from sklearn.cluster import KMeans
from sklearn.decomposition import PCA
from sklearn.metrics import adjusted_rand_score, silhouette_score
from sklearn.mixture import GaussianMixture
from sklearn.preprocessing import StandardScaler


parser = argparse.ArgumentParser(description="Reproduce the WISC-R revision analyses and supplementary tables.")
parser.add_argument("input_workbook", type=Path, help="Path to the de-identified WISC-R analysis workbook")
parser.add_argument("--output-dir", type=Path, default=Path("revision_outputs"), help="Directory for CSV outputs")
args = parser.parse_args()

SOURCE = args.input_workbook
OUT = args.output_dir
OUT.mkdir(exist_ok=True)

SUBTESTS = [
    "Genel Bilgi", "Benzerlikler", "Aritmetik", "Yargılama", "Sayı Dizisi",
    "Resim Tamamlama", "Resim Düzenleme", "Küplerle Desen",
    "Parça Birleştirme", "Şifre",
]


def entropy_index(post):
    k = post.shape[1]
    if k == 1:
        return np.nan
    return 1 + np.sum(post * np.log(np.clip(post, 1e-15, 1))) / (len(post) * np.log(k))


df = pd.read_excel(SOURCE, sheet_name="KATILIMCI_SONUÇLARI", header=3)
cohort = df[
    (df[SUBTESTS] > 0).all(axis=1)
    & (df[["Sözel IQ", "Performans IQ", "Toplam IQ"]] > 0).all(axis=1)
].copy()

X = StandardScaler().fit_transform(cohort[SUBTESTS])
pca = PCA().fit(X)
scores = pca.transform(X)
residual = StandardScaler().fit_transform(X - np.outer(scores[:, 0], pca.components_[0]))
ipsatised = StandardScaler().fit_transform(X - X.mean(axis=1, keepdims=True))
datasets = {"Raw": X, "PC1 residual": residual, "Ipsatised": ipsatised}

# Correlation matrix and PCA loadings/weights.
cohort[SUBTESTS].corr().to_csv(OUT / "table_s1_subtest_correlations.csv")

rng = np.random.default_rng(20260929)
random_eigenvalues = np.empty((1000, len(SUBTESTS)))
for b in range(1000):
    permuted = np.column_stack([rng.permutation(X[:, j]) for j in range(X.shape[1])])
    random_eigenvalues[b] = PCA().fit(permuted).explained_variance_

pca_table = pd.DataFrame({
    "Component": np.arange(1, 11),
```

```python
    "Eigenvalue": pca.explained_variance_,
    "Variance_percent": pca.explained_variance_ratio_ * 100,
    "Parallel_mean": random_eigenvalues.mean(axis=0),
    "Parallel_95th": np.quantile(random_eigenvalues, 0.95, axis=0),
})
pca_table["Retained"] = pca_table["Eigenvalue"] > pca_table["Parallel_95th"]
pca_table.to_csv(OUT / "table_s2_pca_parallel_analysis.csv", index=False)

pc_correlations = pd.DataFrame({
    "Comparison": ["PC1-FSIQ", "PC1-VIQ", "PC1-PIQ", "PC2-FSIQ", "PC2-VIQ", "PC2-PIQ", "PC2-(PIQ-VIQ)"],
    "r": [
        np.corrcoef(scores[:, 0], cohort["Toplam IQ"])[0, 1],
        np.corrcoef(scores[:, 0], cohort["Sözel IQ"])[0, 1],
        np.corrcoef(scores[:, 0], cohort["Performans IQ"])[0, 1],
        np.corrcoef(scores[:, 1], cohort["Toplam IQ"])[0, 1],
        np.corrcoef(scores[:, 1], cohort["Sözel IQ"])[0, 1],
        np.corrcoef(scores[:, 1], cohort["Performans IQ"])[0, 1],
        np.corrcoef(scores[:, 1], cohort["Performans IQ"] - cohort["Sözel IQ"])[0, 1],
    ],
})
pc_correlations.to_csv(OUT / "pca_correlations.csv", index=False)

# GMM comparisons across covariance structures and K=1-10.
gmm_rows = []
for dataset_name, data in datasets.items():
    for covariance in ["full", "tied", "diag", "spherical"]:
        for k in range(1, 11):
            model = GaussianMixture(
                n_components=k, covariance_type=covariance, n_init=10,
                max_iter=1000, reg_covar=1e-6, random_state=42,
            ).fit(data)
            labels = model.predict(data)
            counts = np.bincount(labels, minlength=k)
            silhouette = np.nan
            if k > 1 and counts.min() > 1:
                silhouette = silhouette_score(data, labels)
            gmm_rows.append({
                "Dataset": dataset_name, "Covariance": covariance, "K": k,
                "BIC": model.bic(data), "AIC": model.aic(data),
                "Entropy": entropy_index(model.predict_proba(data)),
                "Silhouette": silhouette, "Smallest_class_n": counts.min(),
                "Smallest_class_percent": 100 * counts.min() / len(data),
            })
pd.DataFrame(gmm_rows).to_csv(OUT / "table_s3_gmm_all_models.csv", index=False)

# K-means separation, 500 80% subsample stability estimates, and raw-profile similarity.
stability_rows = []
profile_rows = []
rng = np.random.default_rng(20260929)
sample_size = round(0.8 * len(X))
for dataset_name, data in datasets.items():
    for k in range(2, 7):
        full = KMeans(n_clusters=k, n_init=50, random_state=42).fit(data)
        ari = []
        for b in range(500):
            idx = rng.choice(len(data), size=sample_size, replace=False)
            resampled = KMeans(n_clusters=k, n_init=10, random_state=1000 + b).fit(data[idx])
            ari.append(adjusted_rand_score(full.labels_[idx], resampled.labels_))
        counts = np.bincount(full.labels_)
        stability_rows.append({
            "Dataset": dataset_name, "K": k,
            "Silhouette": silhouette_score(data, full.labels_),
            "ARI_mean": np.mean(ari), "ARI_median": np.median(ari),
            "ARI_2.5th": np.quantile(ari, 0.025), "ARI_97.5th": np.quantile(ari, 0.975),
            "Smallest_cluster_n": counts.min(), "Smallest_cluster_percent": 100 * counts.min() / len(data),
        })
        if dataset_name == "Raw":
            centroids = np.array([cohort.loc[full.labels_ == j, SUBTESTS].mean().values for j in range(k)])
            correlations = np.corrcoef(centroids)[np.triu_indices(k, 1)]
            profile_rows.append({
                "K": k, "Pairwise_profile_r_mean": correlations.mean(),
                "Pairwise_profile_r_median": np.median(correlations),
                "Pairwise_profile_r_min": correlations.min(), "Pairwise_profile_r_max": correlations.max(),
            })
pd.DataFrame(stability_rows).to_csv(OUT / "table_s4_kmeans_stability.csv", index=False)
pd.DataFrame(profile_rows).to_csv(OUT / "table_s5_profile_correlations.csv", index=False)
```

```python
# Orthogonal bifactor model fitted to the subtest correlation matrix.
S = cohort[SUBTESTS].corr().values
q = len(SUBTESTS)
sign_s, logdet_s = np.linalg.slogdet(S)


def fit_factor_model(model_name, starts=20):
    if model_name == "one-factor":
        factors = 1
        mask = np.ones((q, factors), dtype=bool)
    else:
        factors = 3
        mask = np.zeros((q, factors), dtype=bool)
        mask[:, 0] = True
        mask[:5, 1] = True
        mask[5:, 2] = True

    def unpack(params):
        loadings = np.zeros((q, factors))
        loadings[mask] = params[:mask.sum()]
        uniqueness = np.exp(params[mask.sum():])
        return loadings, uniqueness

    def objective(params):
        loadings, uniqueness = unpack(params)
        sigma = loadings @ loadings.T + np.diag(uniqueness)
        sign, logdet = np.linalg.slogdet(sigma)
        if sign <= 0:
            return 1e9
        return logdet + np.trace(np.linalg.solve(sigma, S)) - logdet_s - q

    best = None
    local_rng = np.random.default_rng(20260929)
    for _ in range(starts):
        initial = np.zeros((q, factors))
        initial[:, 0] = 0.65 + local_rng.normal(0, 0.05, q)
        if factors == 3:
            initial[:5, 1] = 0.25 + local_rng.normal(0, 0.08, 5)
            initial[5:, 2] = 0.25 + local_rng.normal(0, 0.08, 5)
        uniqueness = np.clip(1 - np.sum(initial ** 2, axis=1), 0.1, 0.8)
        result = minimize(
            objective, np.r_[initial[mask], np.log(uniqueness)],
            method="L-BFGS-B", options={"maxiter": 5000, "ftol": 1e-12, "gtol": 1e-8},
        )
        if best is None or result.fun < best.fun:
            best = result
    loadings, uniqueness = unpack(best.x)
    sigma = loadings @ loadings.T + np.diag(uniqueness)
    parameters = mask.sum() + q
    df_model = q * (q + 1) // 2 - parameters
    chi_square = (len(cohort) - 1) * best.fun
    f_baseline = -logdet_s
    df_baseline = q * (q - 1) // 2
    chi_baseline = (len(cohort) - 1) * f_baseline
    cfi = 1 - max(chi_square - df_model, 0) / max(chi_baseline - df_baseline, chi_square - df_model, 0)
    tli = (chi_baseline / df_baseline - chi_square / df_model) / (chi_baseline / df_baseline - 1)
    rmsea = np.sqrt(max((chi_square - df_model) / (df_model * (len(cohort) - 1)), 0))
    residuals = S - sigma
    srmr = np.sqrt(np.mean(residuals[np.triu_indices(q, 1)] ** 2))
    return loadings, uniqueness, sigma, {
        "Model": model_name, "Chi_square": chi_square, "df": df_model,
        "CFI": cfi, "TLI": tli, "RMSEA": rmsea, "SRMR": srmr,
    }


factor_fit = []
for name in ["one-factor", "bifactor"]:
    loadings, uniqueness, sigma, fit = fit_factor_model(name)
    if name == "bifactor":
        total_variance = np.ones(q) @ sigma @ np.ones(q)
        fit["Omega_hierarchical"] = loadings[:, 0].sum() ** 2 / total_variance
        fit["Omega_total"] = sum(loadings[:, j].sum() ** 2 for j in range(3)) / total_variance
        fit["ECV"] = np.sum(loadings[:, 0] ** 2) / np.sum(loadings ** 2)
        pd.DataFrame(
            loadings, index=SUBTESTS, columns=["General", "Verbal", "Performance"]
```

```
        ).assign(Uniqueness=uniqueness).to_csv(OUT / "table_s6_bifactor_loadings.csv")
    factor_fit.append(fit)
pd.DataFrame(factor_fit).to_csv(OUT / "table_s7_factor_model_fit.csv", index=False)

print(f"Analytical cohort: {len(cohort)}")
print(f"Outputs written to {OUT}")
```
